# Internet Searches and Their Relationship to Cognitive Function in Older Adults: Cross-Sectional Analysis

**DOI:** 10.2196/jmir.7671

**Published:** 2017-09-06

**Authors:** Johanna Austin, Kristy Hollingshead, Jeffrey Kaye

**Affiliations:** ^1^ Department of Neurology Oregon Health & Science University Portland, OR United States; ^2^ Florida Institute for Human & Machine Cognition Pensacola, FL United States

**Keywords:** Internet, geriatrics, cognition, executive function

## Abstract

**Background:**

Alzheimer disease (AD) is a very challenging experience for all those affected. Unfortunately, detection of Alzheimer disease in its early stages when clinical treatments may be most effective is challenging, as the clinical evaluations are time-consuming and costly. Recent studies have demonstrated a close relationship between cognitive function and everyday behavior, an avenue of research that holds great promise for the early detection of cognitive decline. One area of behavior that changes with cognitive decline is language use. Multiple groups have demonstrated a close relationship between cognitive function and vocabulary size, verbal fluency, and semantic ability, using conventional in-person cognitive testing. An alternative to this approach which is inherently ecologically valid may be to take advantage of automated computer monitoring software to continually capture and analyze language use while on the computer.

**Objective:**

The aim of this study was to understand the relationship between Internet searches as a measure of language and cognitive function in older adults. We hypothesize that individuals with poorer cognitive function will search using fewer unique terms, employ shorter words, and use less obscure words in their searches.

**Methods:**

Computer monitoring software (WorkTime, Nestersoft Inc) was used to continuously track the terms people entered while conducting searches in Google, Yahoo, Bing, and Ask.com. For all searches, punctuation, accents, and non-ASCII characters were removed, and the resulting search terms were spell-checked before any analysis. Cognitive function was evaluated as a z-normalized summary score capturing five unique cognitive domains. Linear regression was used to determine the relationship between cognitive function and Internet searches by controlling for variables such as age, sex, and education.

**Results:**

Over a 6-month monitoring period, 42 participants (mean age 81 years [SD 10.5], 83% [35/42] female) conducted 2915 searches using these top search engines. Participants averaged 3.08 words per search (SD 1.6) and 5.77 letters per word (SD 2.2). Individuals with higher cognitive function used more unique terms per search (beta=.39, *P*=.002) and employed less common terms in their searches (beta=1.39, *P*=.02). Cognitive function was not significantly associated with the length of the words used in the searches.

**Conclusions:**

These results suggest that early decline in cognitive function may be detected from the terms people search for when they use the Internet. By continuously tracking basic aspects of Internet search terms, it may be possible to detect cognitive decline earlier than currently possible, thereby enabling proactive treatment and intervention.

## Introduction

One in 9 adults over the age of 65 has a diagnosis of Alzheimer disease (AD), the 6th leading cause of death in the United States [1]. Due to the significant public health importance of this disease, many clinical trials have been performed in search of an effective treatment. However, from 2002-2012, the clinical trial success of AD drugs advancing to market was 1 out of 244 tested compounds [2], leading some to call this AD’s “lost decade” [3]. This may in part be due to challenges in early diagnosis of AD. AD is marked by an insidious onset and gradual, subtle decline of cognitive function. By the time cognitive and functional symptoms are detected through clinical assessment, disease progression may already be too advanced for treatment to be most effective. Thus, a major focus of AD research is currently directed to early detection and prevention of the disease [4].

AD is likely the result of the progressive accrual of neuropathological lesions in the brain which ultimately affect multiple aspects of cognition and behavior [5]. Abilities that may be affected by this process are evident in a number of domains that are typically assessed in a clinical evaluation, such as language processing, motor function, and executive functioning. However, assessment during clinic visits may not allow for the detection of subtle changes in real-world behavior that are associated with changes in cognitive function. Furthermore, episodic assessment provides only a snapshot of cognitive function and makes detection of crucial changes difficult. Given that many data points are needed to detect a statistically significant change, this episodic assessment paradigm requires multiple years of data. In contrast, recent work by Dodge et al demonstrated that by collecting more frequent (eg, daily) measures of key variables of interest, it is possible to detect trends and changes in variables over a much shorter period (eg, months instead of years), enabling more sensitive detection of the earliest stages of decline [6]. As subtle changes in everyday function take years to evolve and manifest dementia, the ability to detect these changes through continuous in-home monitoring of everyday behavior and activity holds great promise for early detection of AD [7-10].

One key behavior that may enable real-world identification of daily function is computer use. Engaging with a computer requires motor function to operate the keyboard and move the mouse; language processing to comprehend, select, retrieve, and generate appropriate words; and executive function to plan, inhibit, focus, and shift attention in meaningful and efficient ways. Thus, the way an individual interacts with their computer represents a rich and relatively untapped means of assessing everyday cognition especially among older adults who are at risk of cognitive decline. Notably, although adoption of computers among older adults has lagged younger generations [11], as the baby boomer generation ages, the number of computer-savvy older adults is likely to increase dramatically [12]. Thus, an understanding of the relationship between regular computer use and cognitive function will become highly valuable, especially as 80% of older adults who use the Internet go online at least 3 times per week [12]. This computer monitoring approach has begun to be successfully carried out in older adults by assessing a number of computer use metrics for indexing cognitive change. These include general time-use metrics [13] as well as more specific operational aspects, for example, how a person completes an online task or operates a keyboard [14,15] or mouse [7].

One area of particular potential in this regard is inferring aspects of cognitive function and, more specifically, language function through the terms people search for on the Internet. Previous studies on language analysis (not typically done using computer-based monitoring) have indicated that certain key aspects of language decline in neurocognitive disorders such as AD [16]. These language changes are likely due to disruption of regions of the brain responsible for production and encoding of language [17-20]. Importantly, changes in language function have been observed before the clinical diagnosis of manifest dementia [21]. Aspects of language that decline include semantic fluency, picture naming, and phonetic fluency [22]. More recently, researchers have begun using automatic speech detection or recognition (ASR) systems to analyze not only word use but also pauses and speech tempo [23] and have demonstrated that these key aspects of language are sensitive to cognitive decline before other clinical tests may detect the disease [24,25].

All of this recent research has relied on elicited speech or language using a formal testing paradigm. This study bridges the gap between the previous work on inferring cognitive function from computer use and the work regarding the effects of cognitive decline on language by using continuous computer monitoring software to collect samples of language from the terms people search for on the Internet. Search term language differs from spoken language in that it is frequently goal oriented, may use only key words rather than complete sentences, and may also use terms not used in a spoken language such as “df” instead of “definition.” Although a large body of literature has focused on how older adults search the Internet to find health information [26-28], relatively few studies have investigated general aspects of search term language or how they may relate to cognitive function.

The focus of this paper was to determine whether early language changes can be detected from the way people search the Internet during routine, everyday use. In particular, we hypothesize that individuals with more impaired cognitive function will (1) employ fewer unique search terms per search, (2) employ shorter words in their searches, and (3) use less obscure search terms, where obscurity is defined as the inverse of the frequency of searching for a given term across all subjects.

## Methods

### Participants

The participants for this study were recruited from two ongoing projects: the Life Laboratory cohort (Oregon Health & Science University (OHSU) IRB #2765) and the Ambient Independence Measures (AIMs) cohort (OHSU IRB #9944). The focus of these studies is to understand the relationship between daily behavior and health in older adults using home embedded sensing and computing (“smart home”) technologies [9]. Eligibility criteria for both studies included living alone and independently in a house or apartment larger than a studio, a minimum score of 25 on the Mini-Mental State Examination, and a maximum score of 0.5 on the Clinical Dementia Rating scale (not demented). Participants were also required to live independently without the need for in-home nursing care or help with daily activities. The minimum age for participation in the Life Laboratory study is 62 years, whereas that for the AIMS cohort is 70 years. Enrollment for the Life Laboratory study began in 2007 and continues on a rolling basis. Enrollment for the AIMs study began in 2014 and closed in April 2016.

In 2015, all active participants from both projects who indicated they used a computer were asked to participate in this additional computer use monitoring study. Participants who agreed to have their computer use monitored received software (WorkTime, Nestersoft Inc) on their personal computers, which records all activities performed on the computer, including terms searched for in search engines such as Google, Yahoo, Bing, and Ask.com. All subjects signed informed consent before participating in any study activity, and the study was approved by the OHSU Institutional Review Board (IRB #2765). A total of 76 individuals agreed to participate. Of these, 54 participants searched in Google, Bing, Yahoo, or Ask.com while the software was installed on the computer. However, 12 of these participants did not search during the 3 months before or after completing their neuropsychological evaluation, and thus their Internet search data could not be used. Thus, a total of 42 participants were included in the final analysis. The demographic characteristics of the participants are shown in Table 1. Those participants who completed a search were younger than those who never completed a search, but they were not statistically significantly different in other demographic characteristics. Among those who completed a search, the average age was 81.1 years (SD 10.5), 83% were female (35/42), and 49% had completed college (25/42). The average cognitive z-score (defined in detail under *Cognitive Function* below) in this cohort was 0.16 (SD 0.56), and one participant had a CDR score of at least 0.5, suggesting mild cognitive impairment.

### Data and Measures

For each participant, demographics (including age, sex, and education) were collected at baseline, clinical assessments were performed annually, and (beginning in 2015) data were collected continuously from the participants’ personal computers using WorkTime. Below is a more detailed description of each of these types of data.

#### Internet Searches

The WorkTime software installed on participants’ computers records the websites visited, the applications used, and the search terms entered when performing Internet-based searches. WorkTime collects data from any search browser on any website such as Target.com and Facebook.com. However, searches on websites other than major search engines do not represent the same type of search query as those in the major search engines. We therefore limited the final dataset to consist only of searches entered in major search engines. A google search revealed that the top search engines are Google, Bing, and Yahoo. We therefore included these three search engines in the dataset. We then reviewed each participant to determine if they frequently conducted searches in other major search engines and found that several participants also conducted searches using Ask.com. We therefore also included searches arising from this search engine in our final dataset.

The final dataset represents an average of 370 days of continuous computer use data (min: 7 days; max: 796 days) from 76 participants. From the time the computer software was installed until the data was pulled for analysis, 54 subjects completed 8565 searches in Bing, Google, Yahoo, and Ask.com, whereas 22 participants with WorkTime installed on their personal computer never completed a search in one of these top search engines during the monitoring period, although some of these participants did conduct searches outside the monitoring period. Because we limited the analysis to the 3 months of Internet search data before or after the in-person cognitive evaluation, only 42 participants who conducted an Internet search during this time were included in the final analyses.

Before analysis, all search terms were cleaned using a 3-step process. First, all unreadable characters were removed from the string of search terms. Such characters include symbols and non-ASCII characters which could not be read or interpreted using standard English text analysis techniques. Where applicable, accented or non-ASCII characters were converted into the ASCII equivalent of the character (eg, “ű” was converted to “u”).

**Table 1:** Demographic characteristics of the participants at baseline.

Characteristics	Participants who completed a criterion search (n=42)	Participants who never completed a search (n=32)	*P* value
Age in years, mean (SD)	81.1 (10.5)	88.9 (6.1)	<.001
Sex, female, n (%)	35 (83)	22 (68.8)	.14
Education in years, mean (SD)	15.5 (2.0)	15.3 (2.5)	.62
Cumulative Illness Rating Scale (CIRS) score, mean (SD)	20.3 (2.6)	20.8 (2.6)	.37
Mini-Mental State Examination (MMSE) score, mean (SD)	29 (1.3)	28.6 (1.7)	.46
Clinical Dementia Rating (CDR) score ≥0.5, n (%)	1 (3)	4 (10.3)	.21
Cognitive z-score, mean (SD)	0.16 (0.56)	0.08 (0.76)	.60

Once these characters were removed, punctuation was also removed, all letters were changed to lowercase, and the complete search was divided into individual, unigram search terms. For example, for the search “Where are cooking classes in Portland?” we first removed the question mark from the end and changed the capital letters in “Where” and “Portland” to lowercase letters. We then divided the entire search into its individual terms: “where,” “are,” “cooking,” “classes,” “in,” and “portland.” After each search was divided into its individual terms, we ran a basic spell checker on each term. Spell checking is necessary as we are interested in understanding how cognitive function relates to variables such as the number of unique terms searched for—a variable that would become over inflated for any participant who regularly misspelled words. This is especially important as spelling may decline with deteriorating cognitive function, potentially masking the true relationship to the generation of the search terms themselves regardless of their correct spelling. To determine whether a word was misspelled, we first tested whether the word appeared in a large corpus of words. If the word did not appear there, we assumed the word was misspelled and endeavored to find the correct spelling. This was done algorithmically by first removing individual letters from the word and testing whether the new word appeared in the large corpus of words. If that did not find a suitable match, we swapped letters that were next to each other in order and tested each newly generated word to see whether it was in the large corpus of words. If we still did not find a match, we added individual letters to the word and tested each new generated word against the corpus of words. If none of these methods found a match, we left the word as is.

The final step in the search term preparation was to stem each word to the root word. For the search example above, this would entail removing the “es” from classes (ie, “classes” becomes “class”) and removing the “ing” from “cooking.” To stem the words, we employed the WordNet lemmatizer that is freely available as part of the Natural Language Toolkit for Python. Unlike other stemming tools, this stemmer first checks WordNet’s expansive dictionary to ensure the stemmed word is an actual word before stemming the suffix.

We calculated three metrics using the cleaned search terms. First, we calculated the average number of unique terms per search, which is defined as the total number of words a participant searched for divided by the total number of searches they performed. Second, we calculated the average length of words searched for as the total number of letters in all words divided by the total number of words searched. These two metrics were computed on a per-subject basis (eg, only data from one subject was used to compute that subject’s average number of unique terms or average word length). Finally, we calculated the average obscurity of the words searched. To compute the average term obscurity, we first calculated the frequency of each searched word across all subjects. Using this frequency, we then calculated the obscurity of each term as the inverse of the frequency. Finally, we calculated each participant’s average term obscurity as the average obscurity score of all words that participant searched for.

#### Cognitive Function

Standardized, detailed clinical data were collected at baseline for all participants and then annually to appropriately characterize the participants [10]. These clinical data cover four important domains: health status, physical function, cognition, and mood. Global cognitive status was assessed using a composite score including z-scores tabulated from two or three representative neuropsychological tests in each of five cognitive domains. Cognitive domains that were assessed include *working memory*: Letter-Number Sequencing (WMS-III) [29] and Digit Span Backward length (WAIS-R) [30]; *attention/processing speed*: Digit Span Forward length (WAIS-R), Digit Symbol (WAIS-R), and Trail Making Test Part A [31]; *memory*: WMS-R Logical Memory II Story A, WMS-R Visual Reproduction II, and CERAD Word-List Recall [32]; *executive function*: letter fluency (CFL), Trail Making Test Part B [31], and Stroop color-word conflict [33]; and *visual perception/construction*: WAIS-R Block Design, WAIS-R Picture Completion, and WMS-R Visual Reproduction I. Individual test z-scores were calculated using group mean and standard deviations of the raw test scores from all cognitively intact participants at study entry into the ORCATECH cohorts. All individual participant scores were z-normalized, summed, and averaged to obtain a composite score. This latter score represents global cognitive function, hereafter referred to as the cognitive z-score.

#### Covariates

We included several variables in all models that might confound the relationship between Internet-based search terms and cognitive function. These included age, sex, and years of education, as these are the variables that may relate to cognitive decline.

### Data Analyses

We first computed descriptive statistics for all variables. These included the average number of searches conducted on individuals, the average number of words per search, the average number of letters in each word searched for, and the average obscurity of the terms searched.

Next, we ran 3 linear regressions, each with cognitive z-score as the outcome variable. The first linear regression modeled the relationship between cognitive function and number of unique search terms entered per search. The second regression modeled the relationship between the average length of words searched for and cognitive function. The third regression modeled the relationship between cognitive function and average term obscurity. To ensure coefficient estimates were not biased by multicollinearity, the variance inflation factor (VIF), a standard diagnostic tool for assessing the level of collinearity of the independent variables, was computed for all independent variables. The VIF for all variables was below 2.5, indicating any bias from multicollinearity can reasonably be ignored [34]. All regressions controlled for age, sex, and years of education were performed in Stata 13 (Stata Corp) using the function “reg.”

## Results

### Descriptive Statistics

Participants conducted a median of 22 (interquartile range, IQR 7.3) searches over the 6 month monitoring period (Table 2). The most common terms searched for were “Portland” (n=318 searches), “Oregon” (n=175 searches), “how” (n=40 searches), and “email” (n=36 searches). The mean number of words per search was 3.08 (SD 1.6), and the longest search contained 22 words. Across all words searched for, participants averaged 5.77 (SD 2.2) letters per word. The average term obscurity across participants was 0.25 (SD 0.1).

**Figure 1:**
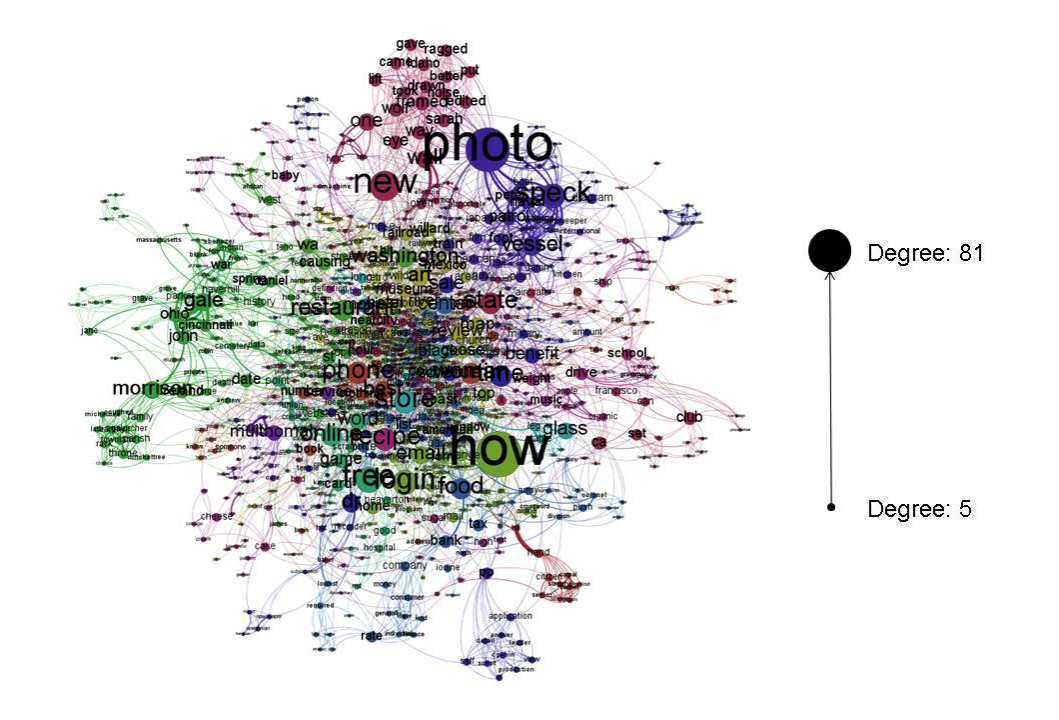
A social network diagram of participant searches over the past year. Search terms are connected to each other if they appeared in the same search, and stronger connections indicate they appeared more frequently together. Each term is sized by the degree of the node, which represents the number of unique terms that are connected to that term. Terms are colored by community, where terms that are frequently searched for together are grouped into the same community.

The richness of the search term dataset is demonstrated in Figure 1 where searches are represented in a social network graph. The figure was created in Gephi 0.8.2, an open source software designed for visualizing social network diagrams. In this figure, each unique term a participant searched for is represented by an individual node, and nodes are joined together if they appeared in the same search (thicker edges indicate they appeared together more frequently). Nodes were sized by their degree such that larger nodes had more unique connections and were colored to represent “communities,” where the communities were determined using Gephi’s modularity function with a resolution of 1.0. The terms ‘Portland” and “Oregon” were searched for so frequently that they overshadowed the rest of the terms and were therefore removed from the graph to allow better visualization of the rest of the network. From the graph, it is clear that people frequently search for “photo” and ”how” which may indicate that people are using the Internet to see pictures of things and to determine how to do things.

**Table 2:** Descriptive statistics of the variables included in the model.

Variables	Statistic	Range (min-max)
Number of searches, median (IQR, interquartile range)	22 (7.3)	(1-718)
Words per search, mean (SD)	3.08 (1.57)	(1-22)
Letters per word, mean (SD)	5.77 (2.23)	(1-28)
Word obscurity, mean (SD)	0.25 (0.11)	(0.52-0.04)

### Linear Regression Analyses

The results of all three linear regressions are presented in Table 3. Note that in this table, the beta coefficients represent the amount cognitive z-score will change for a unit change in each independent variable. For all outcome variables, we present one-sided *P* values commensurate with the directional relationships hypothesized before running the models.

**Figure 2:**
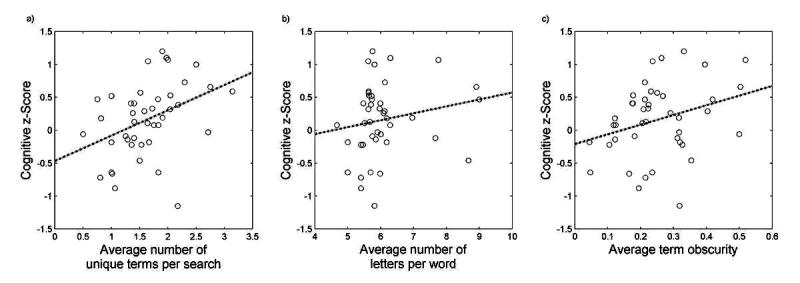
Scatter plots of the relationships between cognitive function and (a) average number of unique terms per search, (b) the average number of letters per word, and (c) the average term obscurity. The observed regression line for each relationship is also plotted as a dashed line.

In the first model, we tested whether the higher cognitive function was associated with more unique search terms entered per search. As shown in Table 3, our results supported this hypothesis: for each additional unique word searched for per search, participants score 0.39 points higher on their cognitive z-score (*P*=.002). To put this in perspective, with this beta coefficient, the model would predict that the participant who averaged the most unique terms per search of 3.1 terms would score 1.01 points higher on their cognitive z-score as compared to the individual who averaged the least unique terms per search of 0.5 terms. This difference is significant considering the range of cognitive z-scores is from −1.15 to 1.2. The R^2^ for this model was 0.46. A scatter plot of the relationship between cognitive z-score and the average number of unique words per search can be visualized in Figure 2.

In our second model, we tested whether the higher cognitive function was associated with using longer words when searching. As shown in Table 3, our results did not support this hypothesis (*P*=.21). Although direction of the coefficient was in the hypothesized direction, the relationship was not significant in this cohort. The R^2^ for this model was 0.25. A scatter plot of the relationship between cognitive z-score and the average number of letters per word can be visualized in Figure 2.

In our final regression, we tested whether individuals with a higher cognitive function would use more obscure words when they searched the Internet. As shown in Table 3, our results supported this hypothesis: for each additional unit increase in the average obscurity of the words searched for, participants scored 1.39 points higher on their cognitive battery (*P*=.02). To put this in perspective, with this beta coefficient, the model would predict that the participant with the highest average term obscurity of 0.52 would score 0.66 points higher on their cognitive z-score compared to the participant with the lowest average term obscurity of 0.044. The R^2^ for this model was 0.32. A scatter plot of the relationship between cognitive z-score and the average term obscurity can be visualized in Figure 2.

In all models, age was significantly related to cognitive function such that older individuals had a lower cognitive function. Sex and education were not significantly associated with cognitive function in any model.

**Table 3:** Results of the three linear regressions relating Internet searches to cognitive function.

Characteristics	Model 1	Model 2	Model 3
	Beta coefficient (SD)	Beta coefficient (SD)	Beta coefficient (SD)
Constant	.75 (0.96)	1.24 (1.10)	1.53 (0.98)
Age	−.024 (0.007)^a^	−.024 (0.008)^b^	−.024 (0.007)^c^
Sex (Female)	.27 (0.20)	.19 (0.23)	.136 (0.22)
Education	.016 (0.038)	.006 (0.043)	.005 (0.041)
Number of Unique Terms per Search	.39 (0.13)^b^		
Average Number of Letters per Word		.084 (0.806)	
Average Term Obscurity			1.39 (0.68)^d^

^a^*P*=.001.

^b^*P*=.004.

^c^*P*=.002.

^d^*P*=.02.

## Discussion

### Study Overview

In this study, we used WorkTime to continuously monitor computer use in a sample of 74 older adults. WorkTime records the terms people search for whenever they conduct an Internet-based search. Using the search terms data from 42 subjects who completed at least one search during the 6 months surrounding (3 months before or 3 months after) a cognitive evaluation, we demonstrated cognitive function is tied to both the average number of unique terms entered per search and obscurity of the searched words. These results present the first time to our knowledge that a continuous aspect of language use, Internet search terms, has been related to the cognitive abilities of older adults. A follow-up analysis should assess this relationship longitudinally in presymptomatic older adults to determine whether continuous assessment of Internet search terms can be used to identify individuals who will eventually transition to mild cognitive impairment.

### Principal Findings

Our first hypothesis was that individuals with a superior cognitive function would employ more unique terms in their searches. Our results supported this hypothesis, the results of which might be considered consistent with the lexical or generative fluency needed to perform well in the standard psychometric tasks of category and phonemic fluency. Previous studies have established that phonemic and category fluency test performance decline with a change in cognitive function [18,19] including transitions leading to Mild Cognitive Impairment [35]. Category fluency represents the ability to name members of a category (eg, animals), whereas phonemic fluency represents the ability to name words that begin with a certain letter. Both are typically assessed by having participants name as many objects or words as possible in a fixed period of time. Due to the close relationship between cognitive decline and language use, these tests are often part of standard batteries of cognitive tests designed to detect MCI or Alzheimer disease. One may consider the task of generating search terms to draw upon similar cognitive resources, and thus the analysis of search terms generated over time and presented here represents the first time this measure of fluency (fluency of Internet search terms or “FIST”) has been linked with cognitive function.

Our second hypothesis was that individuals with a superior cognitive function would search using longer words. However, our results did not support this hypothesis. This is also consistent with previous studies that have found that word length is not as closely related to picture naming ability as term obscurity [18]. That is, vocabulary in Alzheimer disease may decline disproportionately with word obscurity or word familiarity rather than word complexity. Indeed, our final hypothesis was that individuals with a superior cognitive function would search using more obscure search terms. Our results supported this hypothesis independent of years of formal education, which is consistent with previous work linking the decline in vocabulary to cognitive function [23].

### Limitations

This study has several limitations. Of note, the participants included in this study were primarily white, well-educated, and relatively healthy older adults. The results reported here may not generalize to other populations. The sample size was also small, therefore we controlled for only a small number of variables. Future studies should investigate whether variables such as social network size, social economic status, computer fluency, or medication use have any effect on the results reported here. Computer fluency may be especially important as there could be significant differences in computer use not due to cognitive decline but due to familiarity and exposure to the computer [36].

In addition, we limited the search terms included in the model to only those arising from four major search engines: Google, Bing, Yahoo, and Ask.com. Although these are the primary search engines used by these older adults, it is possible that not all searches were captured as some participants may search the Internet using other, less common search engines.

We also had no way to determine whether participants used an autofill to auto complete their searches. Several search browsers provide the option to give suggestions on the potential remaining terms in a search, typically using popular search queries from both the participants and the greater public to inform the suggestions. If participants were using such software when performing searches, it would inject artificial noise into the search terms dataset. Future studies should verify the results presented here in a dataset where the auto complete function was disabled in all participant browsers.

We employed a basic stemmer and spell checker. These utilities ensured that conjugates of words (for example “running” is a conjugate of “run”) would not be counted twice in the term frequency dictionary, and that misspelled words would not be treated as highly obscure terms when they are actually very frequent but misspelled. However, neither of these utilities performed perfectly. For example, while the stemmer correctly stemmed “wolves” to “wolf,” it incorrectly stems “dies” to “dy.” A more sophisticated spell checker and stemmer may enhance future studies.

Our measure of term obscurity was simply the inverse of the frequency with which the word appeared in the search dataset. This was necessary as multiple words such as “Gmail” are common on the Internet but not characterized in common measures of word frequency or rarity. However, because the subject searches were used both to develop the term frequency dictionary and assess the average word obscurity for each subject, it is possible that individuals who searched the Internet more had a lower average term obscurity as their search phrases and terms were entered more frequently into the dataset. Indeed, the number of searches was negatively correlated with the average word obscurity (*r*=−0.23). However, follow-up analysis revealed that the number of searches in the dataset was not related to the cognitive z-score of the individual (*P*=.97). Nevertheless, future studies should normalize the word frequency per subject to compute the relative obscurity of each word.

WorkTime can monitor not only the terms people search for on the Internet but also detailed aspects of computer use such as the time spent in online games or in social websites. Thus, future studies may benefit from assessing the relationship between cognitive function and multiple aspects of computer use, especially as recent studies have demonstrated that the total time spent on the computer and the number of computer sessions is related to cognitive decline [13,37,38]. Variability in computer use has also been linked to cognitive decline [13], but few, if any, studies have assessed more detailed aspects of computer use (eg, total time in online games) and their relationship to cognitive function, especially using an objective monitoring software.

### Conclusions

This work uniquely assessed the relationship between everyday language function as demonstrated through Internet based searches and cognitive function. Several studies have shown a close relationship between language abilities such as vocabulary size, verbal fluency, and semantic ability and overall change in cognitive function. These functions are typically assessed through standardized episodically administered cognitive tests. Previous studies have not been able to assay such cognitive constructs at a level that provides a scalable early detection approach for cognitive decline. This is likely in part due to the lack of methods providing frequent everyday samples of language use. The approach proposed here takes advantage of computer software that makes it possible to continually and unobtrusively capture aspects of language and related complex cognitive activity during routine computer use. In addition, unlike prior work, the data to be analyzed is inherently ecologically valid as it is the individuals’ everyday function that is being assessed. By building on the relationships demonstrated here, it may be possible to develop a system that detects the prodromal stage of Alzheimer disease by continuously monitoring the terms people search for on the Internet along with other aspects of everyday computer use [7,13,14]. This could be accomplished with algorithms that run routinely and securely in the background similar to virus detection software. By developing a naturalistic technique to assess the earliest symptoms of cognitive change, this approach has the potential to significantly advance the diagnostic and assessment process and provide a novel mechanism that can be used in improving the conduct of clinical trials and care for the development of AD treatments. This would have significant and far-reaching effects on older adults experiencing cognitive decline, their families, and the health care system as a whole.

## Abbreviations

AD: Alzheimer disease
CDR: Clinical Dementia Rating
CIRS: Cumulative Illness Rating Scale
MCI: Mild Cognitive Impairment
MMSE: Mini-Mental State Examination
VIF: Variance Inflation Factor
